# Growth of *Brasenia schreberi* requries good water quality and appropriate sediment nitrogen content

**DOI:** 10.3389/fpls.2025.1535395

**Published:** 2025-03-21

**Authors:** Tingfeng Wang, Hangmei Yang, Hongyi Chen, Wei Zhang, Zhenya Liu, Qifan Li, Mei Sun

**Affiliations:** ^1^ Yunnan Key Laboratory of Plateau Wetland Conservation, Restoration and Ecological Services, Southwest Forestry University, Kunming, China; ^2^ National Plateau Wetlands Research Center, Southwest Forestry University, Kunming, China; ^3^ Administrative Bureau of Beihai Wetland Provincial Nature Reserve in Tengchong, Tengchong, China

**Keywords:** aquatic plants, functional traits, sediment nutrients, water quality, ecological response

## Abstract

**Background:**

Stem tissue structures are the basis of stem function and are essential for maintaining the normal physiological metabolism of aquatic plants. Water and sediment conditions are important factors affecting the functional characteristics and physiological metabolism of *Brassenia schreberi*. Due to pollution and other water and sediment issues caused by human activities, the natural habitat and population size of *B. schreberi* have dramatically decreased. Understanding the responses of the functional characteristics of B. schreberi to water and sediment conditions is the key to its scientific conservation and management.

**Objectives and methods:**

This study selected Beihai Wetland in Tengchong, China, which boasts the largest natural habitat of *B. schreberi*, as the research site. To detect the response strategies of *B. schreberi* to water and sediment conditions, the photosynthetic parameters and stem structural characteristics of this species at 17 locations, as well as the water and sediment nutrient parameters at these locations were measured. We examined the relationships between the trait characteristics of *B. schreberi* and the water and sediment parameters by using correlation analysis. The aim was to explore the effects of sediment nutrients and water quality on the photosynthetic and stem structural characteristics of B. schreberi.

**Results and conclusions:**

*B. schreberi* with higher coverage exhibited higher stomatal conductance (*G_s_*) and transpiration rate (*T_r_*), but lower vascular bundle area and ventilation hole area (*P*<0.05), while the net photosynthetic rate (*P_n_*) maintained content, indicating lower utilization efficiency of water and CO_2_. Water temperature (*WT*), sediment nitrogen content (*ω(N)*) and water dissolved oxygen (*DO*) were the main parameters affecting the characters of *B. schreberi*. The *P_n_*, was significantly negatively correlated with *ω(N)*, while it was positively correlated with *DO* and sediment phosphorus content (*P*<0.05). The findings indicate that *B. schreberi* requires good water quality to maintain a high photosynthetic rate and is prone to phosphorus limitation, but it has low requirements for sediment nitrogen content. The findings of this study provide a scientific basis for the habitat restoration and species-specific management of B. schreberi in degraded wetlands.

## 1 Introduction

*Brasenia schreberi*, also known as watershield, is a perennial floating-leaved aquatic macrophyte in the Nymphaeaceae family (also listed in the Cabombaceae family) (Li et al., 2021, 2018). It has a wide yet and sporadic geographical distribution in temperate and tropical regions of Asia, Africa, Australia, North and South America and India (Drzymulska, 2018; Kim et al., 2012). Floating-leaved plants, such as *B. schreberi*, usually plays as a pioneer in the formation of plant communities in many freshwater ecosystems owing to its robust ability for vegetative propagation, and is also a dominant species in areas where the water level is too deep for emergent plants to grow well (Yang et al., 2020; Grasset et al., 2015; Bornette and Puijalon, 2011). It also plays an important role in water purification and other ecological functions. Additionally, *B. schreberi* is a traditional aquatic vegetable in Asia and is also a traditional medicinal plant in China, with high economic value for both edible and medicinal uses (Ran et al., 2020; Wang et al., 2017). However, the natural habitats suitable for the growth of *B. schreberi* are being seriously lost, the distribution range is narrowing, and the numbers of natural populations are decreasing sharply due to excessive harvesting, environmental pollution and climate change, thus many countries have listed it as an endangered and rare species for priority protection (Yang et al., 2020; Li et al., 2018; Xie et al., 2018). The natural *B. schreberi* is officially listed as a national key protected wild plant by the State Council of China in 1999, and is currently listed as a national level II protection of wild plants (Li et al., 2021). Natural populations of *B. schreberi* in China are mainly growing in unpolluted freshwater ponds, lakes, swamps, and even wild farmlands in the provinces of Yunnan, Hunan, Hubei, Jiangxi, and Taiwan etc. In the biodiversity-rich Yunnan region, natural populations of *B. schreberi* are only found in Tengchong Beihai wetland, where hosts the largest wild *B. schreberi* population area in China, covering 100 hectares, which is greater than the combined area of natural *B. schreberi* populations in other regions of China. *B. schreberi* in the Beihai wetland can form pure populations and serves as an important pioneer and dominant species, playing a significant role in the ecological functions of the wetland. Given the current conservation status of the species, the adaptive mechanisms of its natural populations are key scientific issues that need to be thoroughly investigated.

Plant functional traits can effectively regulate the functional response of plants to environmental changes since they are highly sensitive to these changes, and thus they are often used to explore the adaptation mechanisms of plants to the environment (Cheng et al., 2022; Liu et al., 2021). Nevertheless, previous studies have predominantly concentrated on the responses of leaf functional traits to environmental conditions and their role in plant adaptation to environmental changes (such as Islam et al., 2024; Chen et al., 2024; Wang et al., 2022; Slot et al., 2021; Wright et al., 2004), with relatively less attention devoted to the functional traits of other plant structures, such as stems. Stem is the middle part connecting plant leaves and root system. It plays a crucial role in water and material transport, mechanical support, defense, and lodging resistance throughout plant lifecycle (Łoboda et al., 2018; Bociag et al., 2009; Yiotis et al., 2009). The composition structures of stem are generally composed of epidermal structures, vascular bundle structures and stem tissue cells. Stems of wetland plants also possess aerenchyma, which is not found in most terrestrial plants. The stem of *B. schreberi* is composed of primary structures, including the epidermis and mucus, cortex, abscission layer, aerenchyma, and vascular bundles (Yang et al., 2020; Hu et al., 2018). Epidermal structure, covering the surface of all plant organs, serves as a natural protective barrier between plant and external environment (Han et al., 2021). It is composed of thick-walled cells that adhere firmly to each other and exhibit specific mechanical properties that confer the necessary strength for plant growth (Ristic and Jenks, 2002). The epidermis of *B. schreberi* serves as its barrier structure, which is highly sensitive to the growth environment and is a vital component that determines its normal survival (Drzymulska, 2018; Hu et al., 2018). Vascular system is the mechanical support system of higher plants that maintains plant morphology and supports upright growth. It is also the long-distance transport system for water, minerals, and photosynthetic products within plants, which dominates the redistribution and transfer of substances between different parts of plants (Zhang et al., 2022). Parenchyma cells have the function of maintaining plant tissue structure and limiting tissue growth rate (Serrano-Mislata and Sablowski, 2018; Lee, 2018; Brulé et al., 2019). Aerenchyma allows wetland plants to ventilate and store oxygen in hypoxic environments, facilitating the diffusion of oxygen from leaves to the roots and from the rhizosphere to the outside environment, therefor enhancing plant photosynthetic rate and photosynthetic rate (Yang et al., 2020; Seago et al., 2005). The larger the plant’s aerenchyma, the greater the amount of oxygen released from the root tips (Wang and Reid, 2020; Armstrong et al., 2006; Seago et al., 2005). These tissue structures are the basis of stem function and are essential for maintaining the normal physiological metabolism of plants. Stem biomass of *B. schreberi* constitutes the majority of its total biomass, and the physiological functions of stem structural characteristics are of significant importance for its adaptation to different water depths (Zhang et al., 2018). Investigating the environmental response strategies of *B. schreberi* from the perspective of stem functional traits is a crucial part of elucidating its adaptation mechanisms and will significantly contribute to the conservation of the species. While existing research on the stem functional traits of *B. schreberi* has largely focused on the structural composition and performance of its stems, few studies have quantitatively measured these traits and established quantitative relationships with the environment to explore its response strategies to environmental conditions.

Aquatic plants are the primary producers of wetlands and serve as the main transmitters of material and energy flow in lake ecosystems, and they also purify eutrophic water quality (Zhao et al., 2023; Dhote and Dixit, 2009). Additionally, macroaquatic plants play key roles in providing habitat, refugia, and food for biota in shallow lakes (O’Hare et al., 2018). The growth of aquatic plants is influenced by various environmental factors and is an important indicator of ecosystem health status (Dhote and Dixit, 2009). In the context of global environmental changes, more attention are paid to the responses and adaptations of aquatic plants and their ecosystems to climatic conditions at large spatial scales. However, aquatic plants exhibit strong cryptic characteristics that their sensitivity to climatic conditions is often lower than that of terrestrial plants, while they are more sensitive to the microenvironmental conditions. Water and sediment microenvironment conditions among many environmental factors, especially the sediment nutrient conditions, are important factors affecting the functional characteristics of aquatic plants (Pan et al., 2020; Ogdahl and Steinman, 2015). Water quality and sediment nitrogen and phosphorus contents are also the most important environmental factors affecting the growth, reproduction, and survival of *B. schreberi*. Related studies have shown that *B. schreberi* adapts to oligotrophic aquatic environment, and has higher requirements for water quality and sediment nutrients (Drzymulska, 2018; Xie et al., 2018). Habitat protection, especially water environment protection, is crucial for maintaining the population of *B. schreberi* (Chen et al., 2024; Drzymulska, 2018; Xie et al., 2018). A small amount of fertilizer added to the field water can lead to the decay of *B. schreberi* within a few days (Zhang et al., 2015). The permanganate index, total N content, electrical conductivity, and dissolved oxygen content of water, and organic carbon content and total nitrogen content of sediment collectively explained 82.2% of the changes in the mucilage accumulation of *B. schreberi* (Xie et al., 2018). Dissolved oxygen content, nitrogen and phosphorus content and water temperature are the main water factors affecting the leaf economical traits of *B. schreberi*; and the photosynthetic rate is significantly positively correlated with the dissolved oxygen content, ammonium nitrogen content and nitrate nitrogen content of water (Chen et al., 2024). The yield and mucilage thickness of *B. schreberi* are significantly correlated with soil organic matter, total nitrogen, and available nitrogen content (Wang et al., 2020). The aboveground biomass, belowground biomass and numbers of stems and stem nodes of *B. schreberi* are significantly higher in treatments with nitrogen, phosphorus, and organic fertilizers added to the sediment compare to the control group without fertilization (Zheng et al., 2018). Existing research suggests that the growth of *B. schreberi* depends on high-quality water and nutrient-rich sediment, but these claims remain to be thoroughly validated and still require additional supporting evidence. Moreover, the contributions of key sediment nutrients—carbon, nitrogen, and phosphorus—to the growth of *B. schreberi* are not yet well understood.

Based on the above background, how the stem structural characteristics of *B. schreberi* influence its photosynthetic physiological functions, and how these parameters vary along gradients of water environment and sediment nutrients such as nitrogen and phosphorus, are the scientific questions that this study aims to address. This study hypothesizes that the environmental variation within our set of wetlands (both water and sediment characteristics) led to significant variation among stem and photosynthetic traits of *B. schreberi*, the survival of *B. schreberi* require good water quality and nutrient-enriched sediments, and close correlations exist between plant traits and dissolved oxygen content and some pollution indicators of water, as well as the nutrient content of carbon, nitrogen and phosphorus in the sediment. To verify the hypotheses, a study was conducted at Tengchong Beihai wetland, and its natural *B. schreberi* was taken as the research object. The focus of the study was on the important stem nutritional structure of *B. schreberi*, by measuring photosynthetic carbon assimilation parameters as well as stem structural characteristics. This study explores the ecological response strategies of the stem functional characteristics of *B. schreberi* to water and sediment conditions, and verifies the characteristics of water environment and sediment conditions for the growth of *B. schreberi*. This study will provide a case for understanding the functional adaptation strategies of aquatic plants, and provide a theoretical basis for the scientific protection and rational utilization of *B. schreberi*.

## 2 Materials and methods

### 2.1 Study site

The study site is located in the Beihai Wetland Provincial Nature Reserve (N 25°06′42″-25°08′49″, E 98°30′55″-98°35′02″) in Yunnan Province of China. The Beihai Wetland, with a mean altitude of 1,731 m, is surrounded by mountains, exhibiting the characteristics of “basin-lakeside-hillside”. The annual mean temperature is 14.7°C, which is lower than that in most areas with the same latitude and altitude. The annual mean rainfall in the area is 1,750 mm, and the climate is cool and humid. The annual evaporation is 1575 mm, and the annual mean humidity is 79%. According to the Research Report of the Water Quality in 2022, the water in Beihai Wetland is clear, with good water environment conditions. The overall water environment quality category is Class II, and some local spots are Class III. The eutrophication of the water is mild to moderate. The lake is rich in submerged plants such as Hydrilla verticillata, *Utricularia aurea*, and *Myriophyllum* sp*icatum*, as well as floating-leaf plants like B. schreberi and *Trapa incisa*.

The current water surface area of Beihai Wetland is about 300 hm², mainly consisting of two parts: the northern and southern areas. The northern part is the original Beihai Wetland area, with an average water depth of 3 m and a maximum depth of 10 m. The area with a water depth of less than 3 m is sparsely distributed with B. schreberi, with a coverage of about 70%. The northeastern part has a large area of marshy floating mat meadows. The southern part was originally paddy fields reclaimed by farmers along the lake. It was restored to a wetland through a “farmland-to-wetland” project carried out by the local government from 2010 to 2015. The current water depth in the southern part ranges from 0.5 to 2 m. The southwestern part has a large area of overlapping B. schreberi plants, with a coverage reaching 100%. B. schreberi is the dominant species in the southern farmland-to-wetland area of Beihai Wetland and is also the main area for the distribution of this species in Beihai Wetland.

### 2.2 Research material

**Figure 1:**
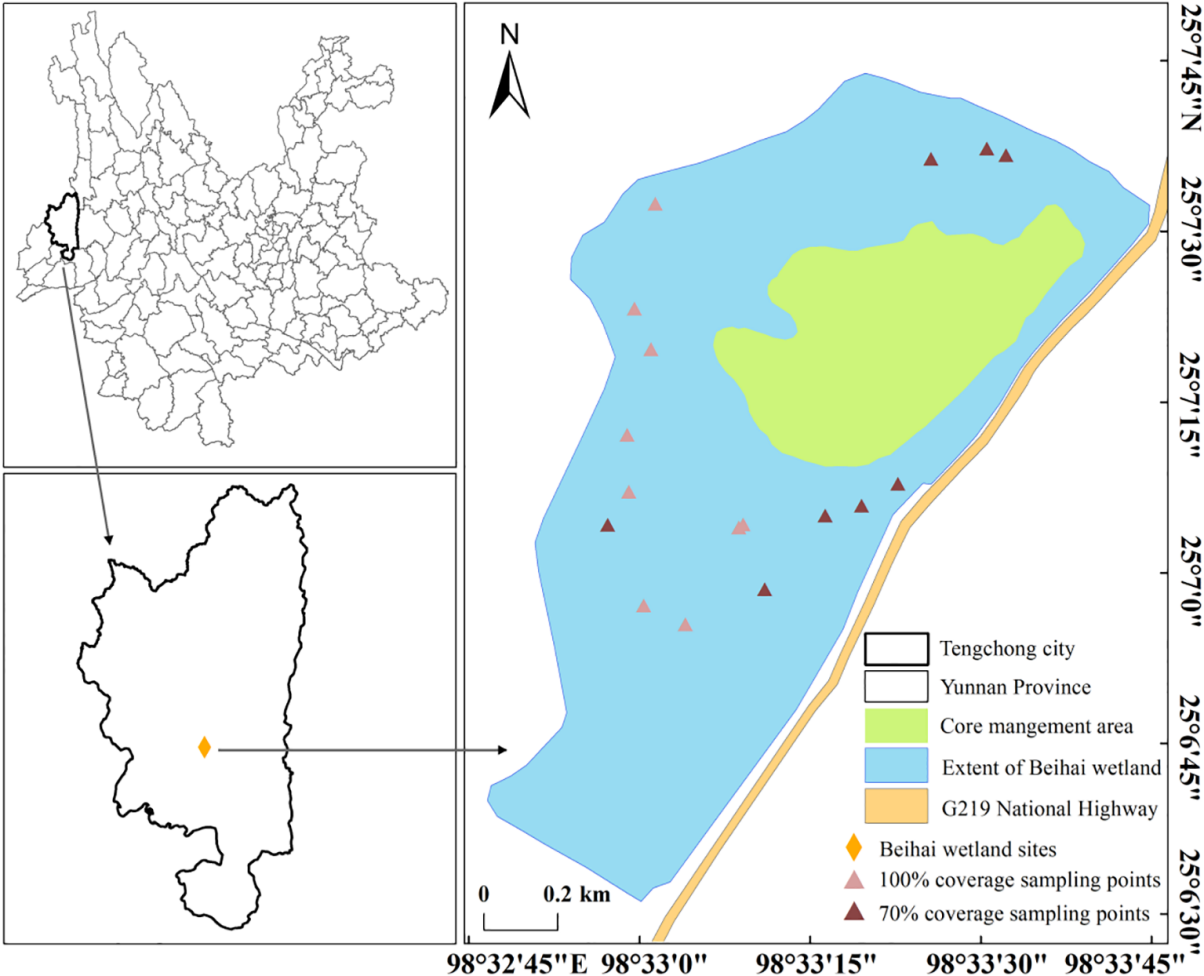
The sampling points were set up in the Beihai Wetland in this study. In the figure, the brownish-red points represent sampling points with a coverage of 70%, which are mainly located in the eastern and northern parts of the wetland, and are sparsely distributed. The pink points represent sampling points with a coverage of 100%, which are mainly located in the southern and southwestern parts of the wetland, and are densely clustered.

According to the field investigation, the growth of *B. schreberi* in Beihai Lake can be roughly divided into two cases. One is that *B. schreberi* is sporadically distributed in the eastern and northern of Beihai Lake, and the coverage of *B. schreberi* is about 70%. Another case is that in the western and the southern parts with the *B. schreberi* is distributed in a large area, and the coverage of the distribution points reaches 100%. According to the ecological environment conditions and the growth and distribution of *B. schreberi*, 17 research points were selected in the distribution range of *B. schreberi* in Beihai Lake, including 8 points with 70% coverage and 9 points with 100% coverage (**Figure 1**). In order to avoid the influence of asexual reproduction of *B. schreberi*, five healthy and similarly growing *B. schreberi* were selected as the study objects at each point, and each plant was separated by more than 5 m. To avoid differences in traits caused by variations in water depth, the water depth at all our sampling sites was maintained at around 1.5 meters, as this depth is the optimal growth depth for B. schreberi (Zhang et al., 2018).

### 2.3 Measurement of plant traits

The physiological functions of stems are mainly reflected in the transport of water, substances, and air, as well as providing structural support and protection for the internal tissues of plant. In this study, we selected vascular bundles, ventilation holes, and epidermal structural traits that correspond to these functions, as well as photosynthetic parameters that can directly reflect the physiological performance of these structures, for quantitative measurement. In June 2022, the field photosynthetic parameters were measured during the peak growth period of *B. schreberi*, and the daily measurement period was between 9:00-12:00 a.m. The net photosynthetic rate (P_n_, μmol·m^−2^·s^−1^), stomatal conductance (G_s_, mol·m^−2^·s^−1^) and transpiration rate (T_r_, mmol·m^−2^·s^−1^) of healthy and mature leaves were measured and recorded *in situ* using LI-6800 photosynthetic fluorescence measuring instrument (LI-6800, LICOR, Nebraska, USA). Before the determination, a small CO_2_ cylinder was installed and the instrument was preheated for 30 minutes. The CO_2_ concentration in the leaf chamber was set at 420 μmol·mol^-1^. The leaf chamber temperature and the air relative humidity were maintaining natural conditions. The leaf chamber temperature is 25-27°C and the air relative humidity is 75-80%. In the determination, the leaves of *B. schreberi* were first induced by 1800 μmol·m^−2^·s^−1^ light for 2 min to maintain the maximum stomatal conductance, and then the light intensity was adjusted to 1500 μmol·m^−2^·s^−1^, and the photosynthetic parameters were determined after the leaf chamber CO_2_ concentration was matched and balanced.

**Figure 2:**
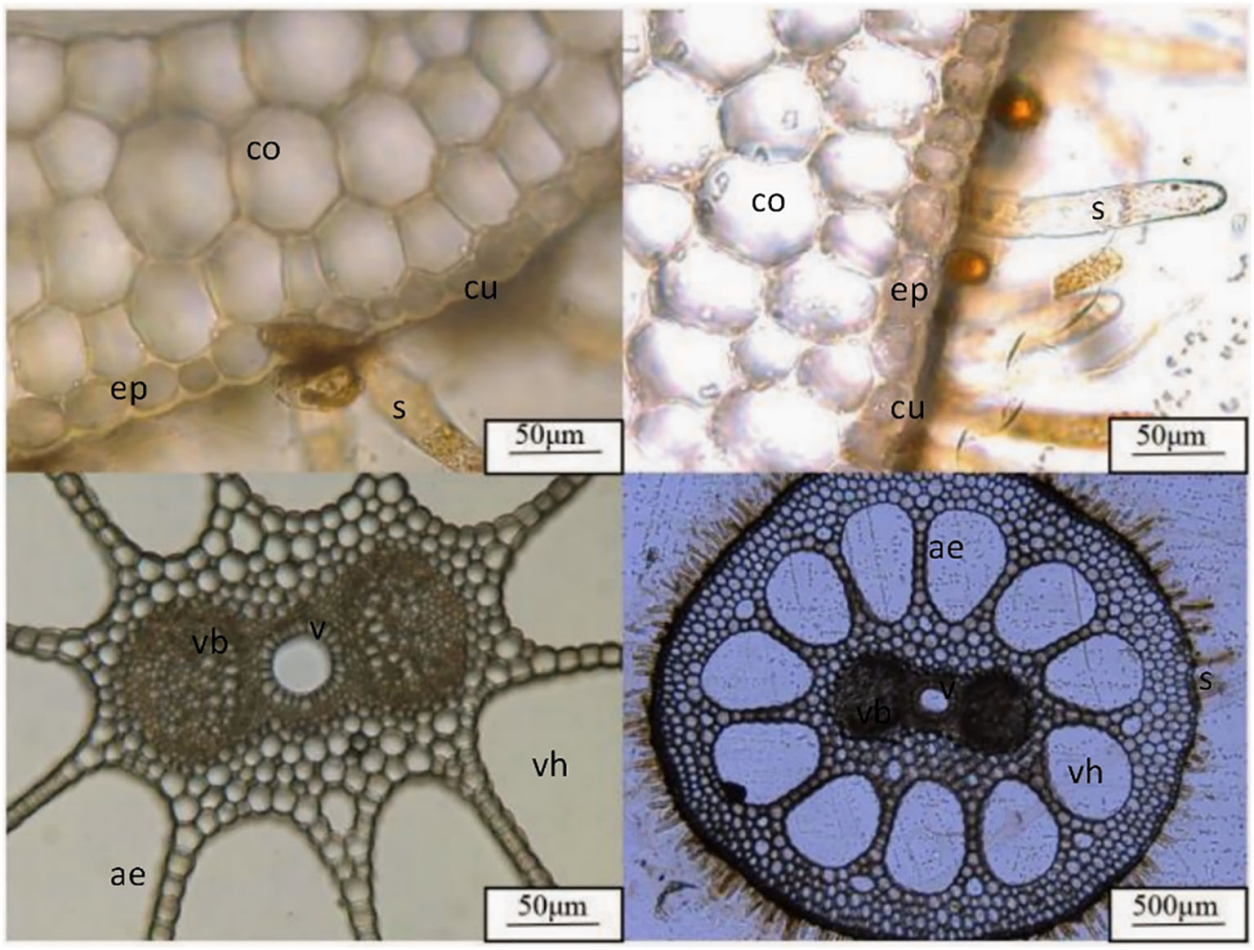
Stem anatomical structures of B. schreberi. co, cortex; ep, epidermis; cu, cuticle; vb, vascular bundle; v, conduit; ae, aerenchyma; vh, ventilation hole; s, mucilage hair.

After measuring the photosynthetic physiological parameters, cut off a section of the stems of the *B. schreberi* that is about 5 cm long, and about 50 cm away from the leaves. Mark the sections well and immerse them in FAA fixative solution (volume ratio of 70% alcohol, 100% glacial acetic acid, and 38% formaldehyde is 90:5:5) for at least 48 hours. After preservation in the preservation box, they were brought back to the laboratory to determine the anatomical structure of the stem sections. In the laboratory, the cross section of the stem of *B. schreberi* was sliced with double-sided stainless blade, stained with 1% toluidine blue for 1 min, and made into temporary water. The slices were observed and photographed under an optical microscope (**Figure 2**). Photographs of epidermal cells and cuticle were taken in the epidermis; then avoid the epidermis and take photos of the vascular bundle structure and the ventilation hole in the middle part. The vascular bundle area (BA, μm^2^), ventilation hole area (VA, μm^2^), cuticle thickness (CT, μm) and epidermal thickness (ET, μm) of the stem were measured and counted by Image J processing software (http://rsb.info.6nih.gov/ij/). The methods for measuring photosynthetic and stem structural parameters have been refined and perfected through our long-term use, and they are now well-established and capable of meeting the requirements for trait measurement.

To minimize the impact of asexual reproduction in *B. schreberi*, a spacing of at least 5 meters should be maintained between each plant during sampling. At each sampling point, five plants are selected to measure functional traits. To ensure robust statistics, when measuring field photosynthetic parameters, an indefinite number greater than five plants (usually 8 to 10) are chosen for measurement. This approach stabilizes the values of most photosynthetic parameters. After discarding the abnormally high and low values, five stable photosynthetic parameters are selected. The plants corresponding to these five parameters are then recorded for photosynthetic traits and subsequently measured for stem structural parameters. Six values were counted for each trait of each plant to ensure that each trait of each study point had 30 statistical values.

### 2.4 Measurement of the sediment element contents

Sediment nutrients are essential for the growth of floating-leaved aquatic plants and significantly impact their growth, ecological functions, and water purification capabilities. Carbon, nitrogen, and phosphorus—macronutrients—are particularly critical, as they determine the metabolic processes and overall growth of these plants and are central to research. Carbon forms the backbone of organic compounds in plants, providing the energy and material basis necessary for their growth and development. Nitrogen and phosphorus, on the other hand, are integral components of many vital organic compounds and directly engage in processes such as photosynthesis, respiration, and energy transfer. They also serve as key constituents of numerous intermediates in photosynthetic metabolism. In this study, a total of 1 kg of sediment samples were collected at each sampling point using a fixed-depth peat drill (Eikel Kampala 0423SA, Netherlands). The samples were then brought back to the laboratory and allowed to air dry naturally. After drying, the samples were finely ground using a soil crusher and sieved through a 100-mesh screen before being sealed and stored. These sediment samples were sent to a third-party testing agency at the Xishuangbanna Tropical Botanical Garden, Chinese Academy of Sciences, to determine the mass fractions (ω, g·kg^-1^) of carbon (C), nitrogen (N), and phosphorus (P) elements.

### 2.5 Determination of water parameters

Besides the sediment nutrient conditions, water quality is another crucial microenvironmental factor influencing the growth of *B. schreberi*. Key indicators for assessing water quality include water temperature (WT, °C), pH value (pH, mol·L^-1^), dissolved oxygen content (DO, mg·L^-1^), nitrogen and phosphorus concentrations, biochemical oxygen demand (BOD, mg·L^−1^), five-day chemical oxygen demand (COD, mg·L^−1^) and potassium permanganate index (CODMn, mg·L^−1^). WT is one of the most basic parameters in water quality monitoring, directly affecting the living conditions of aquatic organisms, the amount of dissolved oxygen, and the reaction rates of chemical substances. The pH, which measures the strength of water’s acidity or alkalinity, also impacts the solubility and toxicity of chemical substances in water. DO is fundamental for the respiration of aquatic organisms and is directly related to their survival. The nitrogen and phosphorus content in water is an important indicator for evaluating the degree of eutrophication. Excess levels of these nutrients can lead to the rapid growth of planktonic plants, causing water turbidity and negatively affecting the growth of large aquatic plants. BOD, COD, and CODMn also reflect the amount of organic matter in water, which is associated with the degree of eutrophication. The DO, pH and WT were measured *in situ* at each sampling point using a multi-parameter water quality analyzer (YSI 650 MDS). Subsequently, 500 mL of water was collected from each sampling point and brought back to the laboratory. In the laboratory, 40 mL of water from each sampling point was filtered and then analyzed using a continuous flow analyzer (Germany SEAL Analytical AA3) to determine and calculate the total nitrogen volume fraction (N_water_, mg·L^−1^) and total phosphorus volume fraction (P_water_, mg·L^−1^). The remaining water samples were sent to a third-party professional testing institution to determine the ammonia nitrogen volume fraction (NH_4_^+^, mg·L^−1^), nitrate nitrogen volume fraction (NO_3_^−^, mg·L^−1^), BOD, COD and CODMn.

### 2.6 Data analysis

The data from this study were analyzed using the statistical analysis softwares of SPSS (v.25, https://spss.en.softonic.com) and Canoco (5.0 https://www.canoco5.com). The data were firstly tested for normality, and the results showed that the data followed a normal distribution. To compare the differences in the functional traits of *B. schreberi* at two different canopy coverages, the independent samples *t*-test was employed with a significance level of *P*<0.05, and the homogeneity of variances between the two groups of data were tested using Levene’s test. Pearson correlation analysis was used to detect the biovariable correlations between the functional traits of *B. schreberi* and environmental condition factors with a significance level of *P*<0.05. Principal component analysis (PCA) of the functional traits of *B. schreberi* revealed that the total variance was less than 3, and then Redundancy analyses (RDA) were conducted to further identify the key water and sediment factors that influence the functional traits and the relationships between the traits and environmental factors.

## 3 Results

### 3.1 Differences in characteristics under two coverages

**Figure 3:**
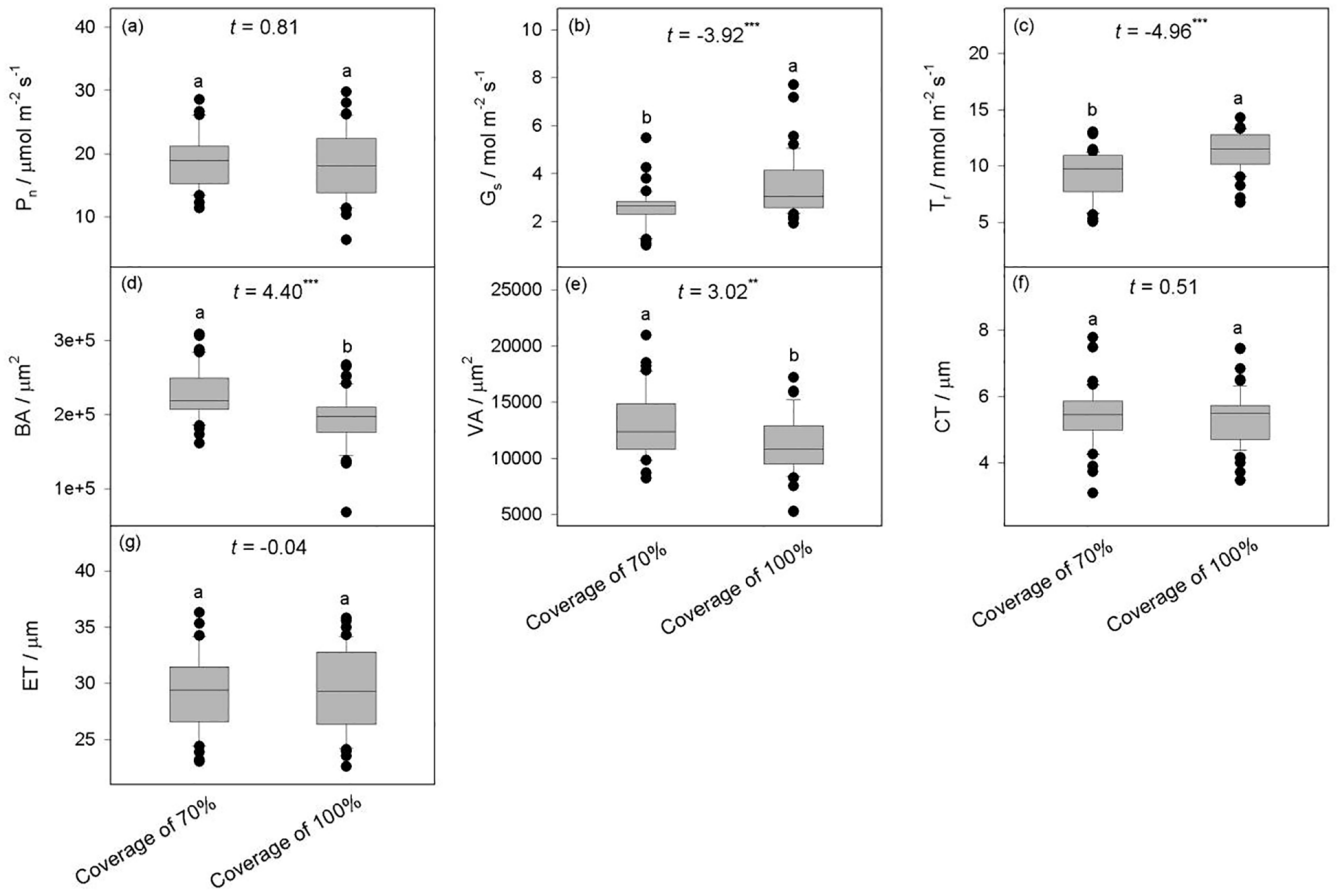
Differences in the characteristics of *B. schreberi* between two canopy coverage. **(a–c)** showed the differences in photosynthetic parameters that higher G_s_ and T_r_ were observed under the 100% coverage, while there was no significant difference in P_n_ between the two coverage. (**d–g**) showed the differences in stem structural characteristics that smaller BA and VA were detected under the 100% coverage, while no differences detected in CT and ET between the two coverage. P_n_, net photosynthetic rate **(a)**; G_s_, stomatal conductance **(b)**; T_r_, transpiration rate **(c)**; BA, vascular bundle area **(d)**; VA, ventilation hole area **(e)**; CT, cuticle thickness **(f)**; ET, epidermal thickness **(g)**. **, *P*<0.01; ***, *P*<0.001.

Compared to *B. schreberi* with a coverage of 70%, the species with 100% coverage exhibited significantly higher stomatal conductance (*G_s_*) and transpiration rate (*T_r_*), but no significant difference were observed in net photosynthetic rate (*P_n_*) between the two coverage (*P*>0.05) (**Figure 3**), indicating that stronger stomatal exchange of water and vapor, and higher leaf water transpiration exhibited in *B. schreberi* with 100% coverage, yet its net photosynthetic rate remains stable, compared to it with 70% coverage. *B. schreberi* with 100% coverage exhibited significantly lower vascular bundle area (*BA*) and ventilation hole area (*VA*) (*P*<0.05), while no significant differences were observed in cuticle thickness (*CT*) and epidermal thickness (*ET*) between the two coverage (*P*>0.05) (**Figure 3**), indicating that plant under 100% coverage showed lower water and air transportion, while its mechanical resistance of the epidermal structure remains stable.

### 3.2 Impact of environmental factors on plant traits

**Figure 4:**
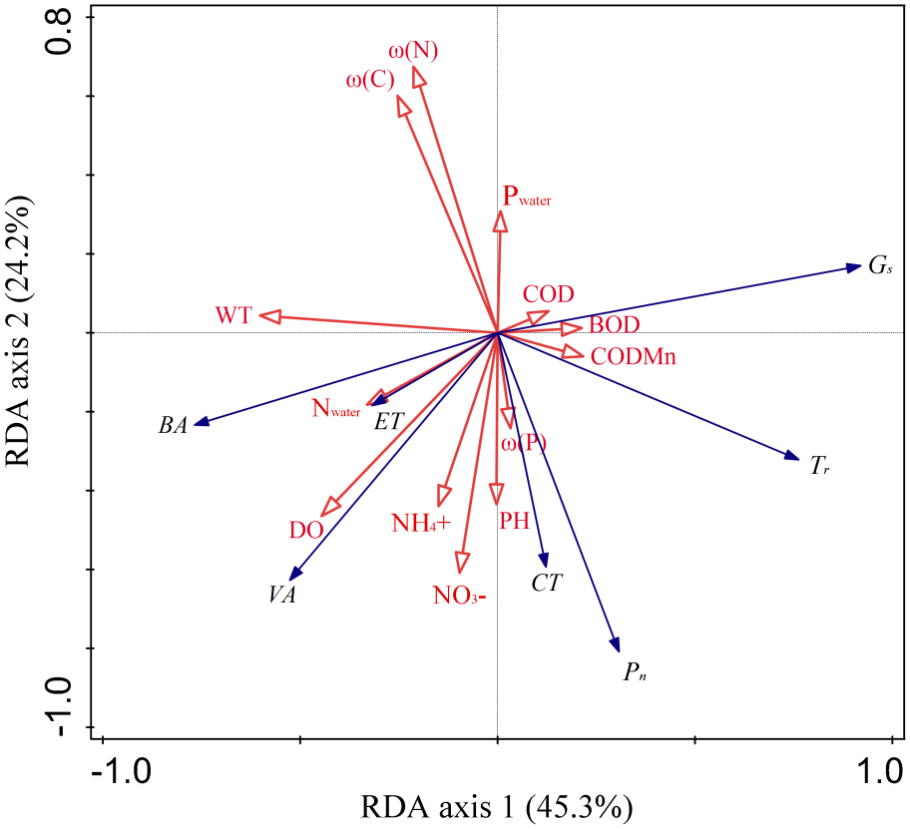
The first two axes of RDA between plant functional traits and environmental factors. The first two axes of *RDA* explained 69.5% of the total variance variation. The six functional traits are all located far from the origin, indicating high sensitivity to water and sediment parameters. Among them, G_s_, T_r_, BA, and VA were mainly distributed along the first axis, while P_n_ and CT were primarily distributed along the second axis. Among the environmental parameters, WT, and DO were mainly distributed along the first axis, while ω(N) and ω(C) were primarily distributed along the second axis. P_n_, net photosynthetic rate; G_s_, stomatal conductance; T_r_, transpiration rate; BA, vascular bundle area; VA, ventilation hole area; CT, cuticle thickness; ET, epidermal thickness. ω(C), sediment mass fraction of carbon; ω(N), sediment mass fraction of nitrogen; ω(P), sediment mass fraction of phosphorus; DO, dissolved oxygen content; pH, water pH value; WT, water temperature; N_water_, water total nitrogen volume fraction; P_water_, water total phosphorus volume fraction; NH_4_^+^, ammonia nitrogen volume fraction; NO_3_^−^, nitrate nitrogen volume fraction; BOD, biochemical oxygen demand; COD, five-day chemical oxygen demand; CODMn, potassium permanganate index.

A redundancy analysis (*RDA*) was used to detect the impact of environmental factors on plant traits. The first two axes of *RDA* explained 45.3% and 24.2% of the total variance variation, respectively (**Figure 4**). *WT*, *ω(N)* and *DO* were the main parameters affecting the characters of *B. schreberi* (**Figure 4**, **Table 1**). *WT* and *DO* mainly negtively correlated with the axis 1, while the *ω(N)* mainly positively correlated with the axis 2 (**Figure 4**). Among the traits of *B. schreberi*, *G_s_* and *T_r_* were mainly positively correlated with the axis 1, while the *BA* and *VA* were mainly negatively correlated with axis 1; *P_n_* and *CT* were mainly negatively correlated with axis 2 (**Figure 4**). Among the environmental parameters, the *WT* has the highest explanatory power, reaching 18.3%; followed by *ω(N)* and *DO* with explanatory powers of 13.1% and 11.8%, respectively (**Table 1**). The explans and contributions of these three parameters have reached significant levels (**Table 1**). The other parameters and their explanatory powers were *ω(P)* 8.4%, *N_water_* 7.8%, *CODMn* 5.2%, *COD* 4.9%, *ω(C)* 4.5%, *P_water_* 4.1%, *NH_4_^+^* 4%, *pH* 3.7%, *BOD* 3.4%, and *NO_3_^-^* 2.1% (**Table 1**). Ranking of the contributions of the environmental parameters is consistent with their explans.

**Table 1:** The explains and contributions of environmental parameters to traits of *B. schreberi*.

Environmental parameters	Explains %	Contributions %	pseudo-F	*P*
Water temperature, WT	**18.3**	**20.1**	3.4	**0.014**
Sediment mass fraction of nitrogen, ω(N)	**13.1**	**14.3**	2.7	**0.030**
Dissolved oxygen content, DO	**11.8**	**12.9**	2.7	**0.044**
Water total nitrogen volume fraction, N_water_	7.8	8.5	1.9	0.128
Sediment mass fraction of phosphorus, ω(P)	8.4	9.3	2.3	0.076
Ammonia nitrogen volume fraction, NH_4_^+^	4.0	4.4	1.1	0.364
Biochemical oxygen demand, BOD	3.4	3.7	0.9	0.44
Potassium permanganate index, CODMn	5.2	5.7	1.5	0.202
Water total phosphorus volume fraction, P_water_	4.1	4.5	1.2	0.320
Water pH value, pH	3.7	4.0	1.1	0.390
Sediment mass fraction of carbon, ω(C)	4.5	4.9	1.4	0.28
Nitrate nitrogen volume fraction, NO_3_^-^	2.1	2.3	0.6	0.63
Five-day chemical oxygen demand, COD	4.9	5.4	1.7	0.242

WT has the highest explanatory power, reaching 18.3%; followed by ω(N) and DO with explanatory powers of 13.1% and 11.8%, respectively (**Table 1**). The other parameters and their explanatory powers were ω(P) 8.4%, N_water_ 7.8%, CODMn 5.2%, COD 4.9%, ω(C) 4.5%, P_water_ 4.1%, NH_4_^+^ 4%, pH 3.7%, BOD 3.4%, and NO_3_^-^ 2.1%. Ranking of the contributions of the environmental parameters is consistent with their explans.

The bold fonts in the "Explains %" column indicate that the Explains values for each indicator are greater than 10%; the bold fonts in the "Contributions %" column indicate that the Contributions values for each indicator are greater than 10%; the bold fonts in the "P-value" column indicate that the significance level is less than 0.05.

### 3.3 Correlations between plant characteristics and environmental parameters

With the biovariate correlations between plant traits and sediment elements, the *P_n_*, *T_r_*, *CT* and *ET* were all significantly negatively correlated with *ω(C)* and *ω(N)*, while the *P_n_* was positively correlated with *ω(P)* (*P*<0.05; **Table 2**). This result indicated that excessive carbon and nitrogen in the sediment showed reduce the net photosynthetic rate of *B. schreberi* and thin its epidermal barrier structure, thereby weakening the protective function of the barrier.

**Table 2:** The correlation coefficients (*r*) of plant characteristics to environmental parameters.

	P_n_	G_s_	T_r_	BA	VA	CT	ET
ω(C)	-0.286^**^	-0.105	-0.406^***^	0.039	-0.046	-0.223^*^	-0.238^*^
ω(N)	-0.302^**^	-0.076	-0.400^***^	0.031	-0.097	-0.284^**^	-0.238^*^
ω(P)	0.257^*^	0.123	-0.086	0.194	-0.021	0.031	0.193
N_water_	-0.022	-0.136	-0.182	0.104	0.156	0.195	0.317^**^
P_water_	-0.202	0.096	-0.116	0.040	-0.125	-0.079	0.097
NH_4_^+^	0.329^**^	-0.064	-0.168	0.105	0.240^*^	0.230^*^	-0.077
NO_3_^-^	0.272^*^	-0.155	0.116	-0.051	0.273^*^	0.314^**^	0.051
COD	-0.086	0.075	0.182	-0.111	-0.036	-0.012	0.211
BOD	-0.026	0.126	0.212	-0.162	-0.043	0.019	0.211
CODMn	-0.007	0.080	0.234^*^	-0.166	-0.049	0.041	0.256^*^
DO	0.255^*^	-0.185	-0.239^*^	0.377^***^	0.359^***^	0.122	-0.074
WT	-0.049	-0.192	-0.355^***^	0.473^***^	0.217^*^	-0.135	0.128
pH	0.180	-0.051	0.116	-0.051	0.245^*^	0.288^**^	-0.140

The P_n_, T_r_, CT and ET were all significantly negatively correlated with ω(C) and ω(N), while the Pn was positively correlated with ω(P), suggestting that the photosynthetic productivity and stability of the epidermal barrier structure of *B. schreberi* did not require excessive carbon and nitrogen in the sediment, while its net photosynthetic productivity relied more on the phosphorus in the sediment. For the water parameters, overall, DO, WT, pH, NH_4_^+^, and NO_3_^−^ were mainly positively correlated with P_n_, BA, VA, and CT, indicated that higher levels of these parameters could promote the net photosynthetic productivity of *B. schreberi*, enhance the transport of water and air, and stabilize the cuticle barrier structure.

P_n_, net photosynthetic rate; G_s_, stomatal conductance; T_r_, transpiration rate; BA, vascular bundle area; VA, ventilation hole area; CT, cuticle thickness; ET, epidermal thickness.

ω(C), sediment mass fraction of carbon; ω(N), sediment mass fraction of nitrogen; ω(P), sediment mass fraction of phosphorus; DO, dissolved oxygen content; pH, water pH value; WT, water temperature; N_water_, water total nitrogen volume fraction; P_water_, water total phosphorus volume fraction; NH_4_^+^, ammonia nitrogen volume fraction; NO_3_^−^, nitrate nitrogen volume fraction; BOD, biochemical oxygen demand; COD, five-day chemical oxygen demand; CODMn, potassium permanganate index.

The bold fonts indicate significance. Significance level: *, P<0.05; **, P<0.01; ***, P<0.001.

With the biovariate correlations of plant traits and water parameters, The *P_n_*, *VA* and *CT* were all significantly positively correlated with *NH_4_^+^* and *NO_3_^-^* (*P*<0.05; **Table 2**). *P_n_*, *BA* and *VA* were all significantly positively correlated with *DO*, the latter two traits were also both significantly positively correlated with *WT*, while *T_r_* was significantly negatively correlated with *DO* and *WT* (*P*<0.05; **Table 2**). Besides, *T_r_* and *ET* were positively correlated with *CODMn*; *VA* and *CT* were both positively correlated with *pH* (*P*<0.05; **Table 2**). *N_water_*, *P_water_*, *COD* and *BOD* contribute little to the plant characteristics, with most of the correlations were insignificant but the correlation between *N_water_* and *ET* (**Table 2**). Overall, *DO*, *WT*, *pH*, *NH_4_^+^*, and *NO_3_^−^* were mainly positively correlated with *P_n_*, *BA*, *VA*, and *CT*, indicated that higher levels of these parameters could promote the net photosynthetic rate of *B. schreberi*, enhance the transport volumes of water and air, and stabilize the cuticle barrier structure.

### 3.4 Correlations among plant characteristics

Some relations existed in the plant characteristics of *B. schreberi*, reflecting their functional associations. *G_s_* was positively correlated with *T_r_* (**Table 3**), indicates that the process of water and vapor exchange through stomata is coupled with the process of water transpiration loss, and they together regulate net photosynthetic production to maintain stability. Larger *BA* and *VA* can transport more water and air at one time, but they correspond to lower transport efficiency. The *BA*, *VA* and *ET* were significantly positively correlated with each other, and the three traits were all negatively correlated with *G_s_*; *BA* also significantly negatively correlated with *T_r_* (**Table 3**). These results indicated that *B. schreberi*, under higher coverage, exhibited higher stomatal water and vapor exchange and transpirational water loss, corresponding to higher water and air transport efficiency. However, the mechanical stability of the epidermal barrier decreased.

**Table 3:** The bio-variate correlations among plant characteristics.

	*P_n_*	*G_s_*	*T_r_*	*BA*	*VA*	*CT*	*ET*
*P_n_*		0.440	0.269	0.217	0.071	0.072	0.855
*G_s_*	0.085		**0.000**	**0.002**	**0.009**	0.247	**0.005**
*T_r_*	0.121	**0.764**		**0.009**	0.329	0.917	0.085
*BA*	0.135	**-0.325**	**-0.282**		**0.000**	0.383	**0.002**
*VA*	0.197	**-0.283**	-0.107	**0.656**		0.068	**0.032**
*CT*	0.196	-0.127	-0.011	0.096	0.199		**0.014**
*ET*	0.020	**-0.304**	-0.188	**0.330**	**0.233**	**0.265**	

G_s_ was positively correlated with T_r_ indicates a functional association between the exchange of water and vapor and the water transpiration loss. The BA, VA and ET were significantly positively correlated with each other, and the three traits were all negatively correlated with G_s_, indicate that B. schreberi under higher coverage has lower transport volume but higher transport efficiency of water and air, and has lower mechanical stability of the epidermal barrier.

In the table, the below part showed the correlation coefficients (*r*), while the above part showed the significance level values (*P*).

*P_n_*, net photosynthetic rate; *G_s_*, stomatal conductance; *T_r_*, transpiration rate; *BA*, vascular bundle area; *VA*, ventilation hole area; *CT*, cuticle thickness; *ET*, epidermal thickness.

The bold fonts indicate significance.

## 4 Discussion

### 4.1 Differences in characteristics under two coverages

Stomatal conductance and transpiration rate are generally positively correlated with photosynthetic rate since they both reflect the ability of water and CO_2_ exchange during the photosynthetic carbon assimilation, although under some extreme conditions (such as drought, high temperature, or salt stress), plants may respond to stress by closing their stomata, which can lead to a decrease in stomatal conductance and transpiration rate. The *B. schreberi* with 100% coverage had higher stomatal conductance and transpiration rate compared to this species with 70% coverage (**Figure 3**), indicating that *B. schreberi* with higher coverage had higher photosynthetic water vapor and CO_2_ exchange capacity, and higher photosynthetic rate in theory. However, there was no significant difference in the net photosynthetic rate between the two groups, indicating that the higher coverage of *B. schreberi* reduced the utilization efficiency of water and CO_2_, the same photosynthetic capacity needed to consume more water and CO_2_ at higher coverage (Peixoto et al., 2016; Oliveira-Junior et al., 2018; Liu et al., 2024). Even if the increase of water vapor and CO_2_ exchange increased the actual photosynthetic rate of *B. schreberi*, the intense plant respiration under hypoxic conditions would lead to a decrease or no change in photosynthetic rate (Dusenge et al., 2019). The smaller stem vascular bundles and ventilation holes of *B. schreberi* with higher coverage (**Figure 3**), may be a response to higher water vapor and air exchange capacity. Higher water vapor exchange capacity requires faster water and air transport, so higher water and air transport efficiency is required (Pan et al., 2021; Zhao et al., 2022). Small vascular bundles and ventilation holes generally correspond to a greater numbers of the structures, which decrease the risk of cavitation with the increase of velocity in the process of transporting water and air, thus increasing the relative area of transportation and improving the efficiency of water and air transportation (Caraco et al., 2006; Qaderi et al., 2019). Therefore, *B. schreberi* with high coverage will reduce their utilization efficiency of water and CO_2_, while increase their efficiency of water and air transportation, indicating that high coverage exacerbates the habitat stress on *B. schreberi*. This result supports the hypothesis of this study that the growth of *B. schreberi* requires good water quality. Under high cover conditions in Tengchong Beihai wetland, the water where *B. schreberi* grows has significantly lower dissolved oxygen content (DO=1.9 mg/L) and significantly lower water temperature (WT=21.8 ℃) reflecting deteriorated water quality. Previous studies also pointed the high coverage of floating leaves and floating plants will lead to a serious decrease in the amount of oxygen available in the lower layer of the plant (Oliveira-Junior et al., 2018; Henriot et al., 2019). The respiration of the leaves and roots in the lower layer of the plant is strong, and the plant must transport more air to maintain the survival of the plant (Dai et al., 2012; Tang et al., 2017; Oliveira-Junior et al., 2018).

### 4.2 Impact of environmental factors on plant traits

Water and sediment serve as vital sources of nutrients and energy required for the growth and development of aquatic plants, and their environmental conditions significantly influence the expression of functional traits in these plants (Pan et al., 2020; Henriot et al., 2019; Xie et al., 2018). Water temperature and dissolved oxygen content are the most significant water factors affecting the photosynthetic physiology and stem structural traits of *B. schreberi* (**Figure 4**; **Tables 2**, **3**). Floating-leaved plants reduce water temperature and dissolved oxygen content through reducing water transmittance and gas exchange between water and air, which in turn limits water reoxygenation and photosynthetic enzyme activity, respectively, increases photorespiration and plant respiration, and reduces net photosynthetic rate of *B. schreberi* (Jiang et al., 2022; Said et al., 2021). Stable net photosynthetic rate is the foundation to ensure plant normal growth, reproduction, and dispersion under stress conditions (Lamers et al., 2020; Li et al., 2021). Higher transpiration rate and stomatal conductance, along with smaller vascular bundle area and aerenchyma area, are the typical phenotypic traits that contribute to higher photosynthetic rate. Therefore, the net photosynthetic rate of *B. schreberi* may be enhanced by increased water-vapor exchange capacity and improved water and air transport efficiency, thus the photosynthetic limitation caused by low temperatures can be alleviated and stable net photosynthetic rate can be maintained. The significant correlations of stomatal conductance and transpiration rate to vascular bundle area and aerenchyma area, indicating that these four traits play similar roles in maintaining the stability of plant photosynthetic function and close functional relationships exist in the traits. The significant positive correlation between net photosynthetic rate and dissolved oxygen also suggests that a higher oxygen supply is the foundation for ensuring higher photosynthetic rate in plants.

The rhizomes of floating-leaved plants are rooted in the sediment, and obtained nutrients from the sediment, making sediment nutrient conditions an important factor that affects their growth strategies (Henriot et al., 2019; Titus and Gary Sullivan, 2001). Sediment nitrogen content exhibits significant negative correlations with net photosynthetic rate, transpiration rate, epidermal thickness, and cuticular thickness of *B. schreberi* (**Table 2**), indicating that excessively high nitrogen levels in the sediment are not conducive to the photosynthetic production of *B. schreberi*, and also limit the epidermic water retention and mechanical support capabilities of its stem epidermis structure. Nitrogen is a key component of plant chlorophyll, proteins, and some other components (Larson and Funk, 2016). In general, the availability of nitrogen in the environment is a crucial factor determining plant growth, and high levels of environmental nutrients can promote rapid plant reproduction and expansion (Wright et al., 2004). Some former studies also pointed the growth of *B. schreberi* requires good water quality and nutrient-enriched sediments based on mucilage content (Li et al., 2021; Xie et al., 2018), and its artificial propagation often requires applying sufficient base fertilizer before planting (Zhang et al., 2018), while our results are not entirely consistent with this statement based on net photosynthetic rate. The distribution sites in this study are mainly located in areas where farmland has been restored to wetlands and water has been stored, resulting in relatively high levels of nutrient elements, providing sufficient nutrients for the growth of *B. schreberi*. Therefore, this study has, to a certain extent, verified that the growth of *B. schreberi* requires fertile sediment. However, high sediment nitrogen inhibited the photosynthetic physiology of *B. schreberi*, thus a limit should be placed on the nitrogen demand of *B. schreberi*, indicating that excessive sediment nitrogen has an inhibitory effect on photosynthetic physiology, while moderate nitrogen content is the optimal condition.

Under hypoxic wetland conditions, nitrogen in the sediment is converted into higher concentrations of ammonium nitrogen through the process of denitrification. Elevated ammonium concentrations typically facilitate the production of various phytotoxic compounds in the rhizosphere, which inhibit the photosynthetic physiological processes of plants and restrict the increase in plant growth characteristics (Ponnamperuma, 1972; Pezeshki, 2001). For instance, high ammonium concentrations and temperatures in wetlands are usually significantly and negatively correlated with the growth and biomass production of rhizomes (Henriot et al., 2019; Klok and van der Velde, 2017). Size parameters are considerded as growth traits, while substances like the mucilage of *B. schreberi*, are primarily secondary metabolites (Henriot et al., 2019). A higher amount of secondary metabolites often incurs greater construction costs, thereby weakening growth traits, and therefore, under high-nitrogen substrate conditions, the accumulation of more mucilage (such as Li et al., 2021; Xie et al., 2018) is a stratage for plants to alleviate stress, but it may not conducive to their good growth and reproduction that the latter are frequently related to plant competitive ability (Grime, 2006). In the conservation and management of rare aquatic plants such as *B. schreberi* in wetland protected areas, environmental conditions should be carefully controlled (for example, by increasing dissolved oxygen and reducing sediment nitrogen content) to ensure that more photosynthetic products are allocated to plant growth rather than to the production of secondary metabolites. Conversely, for artificial cultivation aimed at obtaining higher yields of secondary metabolites, while ensuring the basic growth conditions of the plants, efforts can be made to direct more photosynthetic products towards the synthesis of these secondary metabolites.

Phosphorus is a key constituent of nucleic acids, energy carrier adenosine triphosphate (ATP), and numerous enzymes. It plays a vital role in photosynthetic processes like photophosphorylation and the Calvin cycle, and is essential for cell division and nutrient uptake in plants. Therefore, the supply of phosphorus is crucial for the healthy growth and high yield of plants. The significant positive correlation between sediment phosphorus content and net photosynthetic rate (**Table 2**) indicates that compared to sediment nitrogen, sediment phosphorus content has a stronger limiting effect on the photosynthetic production of *B. schreberi*. Similarly, some growth traits, such as leaf size and stem size also have previously been demonstrated to increase with nutrient content and particularly with phosphates (Henriot et al., 2019; Klok and van der Velde, 2017). Compared to the effects of nitrogen, phosphorus has a greater impact on the growth and reproduction traits of *Nuphar lutea* (a close relative of *B. schreberi)*, with its rhizome size and number of flowers are significantly positively correlated with the phosphorus content in the sediment, while they show negative correlations with the nitrogen content in the sediment (Henriot et al., 2019). In salt marshes, the addition of phosphorus or a combination of nitrogen and phosphorus induces a rapid shift in community dominance from microalgae to higher plants, particularly *Eleocharis* spp. and *Typha domingensis*, and additionally, phosphorus addition results in a four- to five-fold increase in tissue phosphorus content in *Eleocharis* compared to control plants (Rejmánková et al., 2008). In phosphorus-limited wetlands, aquatic plants often show high sensitivity to phosphorus. Even minor additions of phosphorus can spur rapid plant growth and boost photosynthetic rate, thus enhancing their competitive ability. The significant correlation between the photosynthetic rate of B. schreberi and phosphorus in Tengchong Beihai wetland indicates that this wetland is phosphorus-limited.

Epidermal structure is an important barrier in plants and maintaining a healthy epidermal structure is essential for plants to grow and thrive (Yang et al., 2020). Epidermal structure provides protection against external factors such as pathogens, insects, and harsh environmental conditions, while also regulating water loss and gas exchange through its pores, stomata, and waxy coatings (Thompson and Gilbert, 2014). In addition, the epidermal structure also contributes to the mechanical support of the plant body. Thin and discontinuous cuticle of epidermis may contribute to aquatic plants sensitivity to water pollution, like *Genlisea* and *B. schreberi* (Yang et al., 2020; Płachno et al., 2005). The significant negative correlations of sediment nitrogen content to epidermal thickness and cuticular thickness suggests that high sediment nitrogen levels can thin out the cuticle and epidermis of *B. schreberi*, thereby weakening its protective barrier structure. This indicates that while nitrogen is a necessary nutrient for plant growth, excessive nitrogen in the sediment can have negative impacts on the epidermal integrity and function of this aquatic plant (Zhang et al., 2015; Xie et al., 2018).

This study aims to investigate the relationships between the stem structure and photosynthetic traits of *B. schreberi* and environmental variables within a single wetland over a one-year study period. As *B. schreberi* is a long-lived plant species, the relationship between its environment and species traits may occur over a longer timescale than the duration of this study. Similarly, its large rhizomes may integrate responses to environmental signals over a very large scale, potentially leading to an attenuation of the measured responses at the rhizome fragment level, despite previous studies highlighting the reactivity of plant ramets to habitat variability. Although the environmental characteristics of the Tengchong Beihai wetland show some variation, we may still have underestimated the impact of environmental features on plant traits at broader and larger scales. Additionally, the timescale of the study does not allow for the assessment of environmental changes that occur over longer periods. For example, the continuous input of nutrient-rich agricultural water from surrounding areas and increasing biotic competition and invasions within the wetland may have adverse effects on *B. schreberi* over timescales exceeding one year, gradually degrading its health over time. Our study primarily used correlation analysis to detect the close relationships between traits and the environment; however, these relationships still need to be further validated through causal analysis and controlled experimental approaches, which will be the focus of our future work.

## 5 Conclusion

Dissolved oxygen, water temperature, nitrogen and phosphorus contents in the sediment are the primary factors influencing the stem structure and photosynthetic traits of *B. schreberi*. High plant coverage of *B. schreberi* results in decreased water temperature and dissolved oxygen levels. In response, the plant exhibits higher stomatal conductance and transpiration rates, while the size of stem vascular bundles and aerenchyma tissues decreases, the net photosynthetic rate remains constant, indicating reduced efficiency in the utilization of water and CO_2_ and *B. schreberi* requires good water quality for growth. Its high photosynthetic rate does not depend on high sediment nitrogen content but is significantly positively correlated with sediment phosphorus content, suggesting that it is prone to phosphorus limitation. Based on these findings, in the conservation and management of rare aquatic plants like *B. schreberi* in wetland protected areas, it is crucial to control environmental conditions to enhance plant photosynthesis, morphological size, and other growth characteristics, thereby boosting plant competition. Additionally, efforts should be made to reduce the production of secondary metabolites, such as mucilage. For example, increasing dissolved oxygen levels, moderately raising water temperature, and managing sediment nitrogen while increasing sediment phosphorus content are recommended strategies. Expanding the research scope, conducting continuous dynamic studies, and developing models to validate the causal relationships between traits and environmental factors will be key priorities for future work.

## Data Availability

The data analyzed in this study is subject to the following licenses/restrictions: The associated data of this paper are not suitable for sharing but can be obtained from the author upon reasonable request. Please contact the author at sm0510215@163.com.
